# Supplementary material for: Antibiotic use in mandarin production (Citrus reticulata Blanco) in major mandarin-producing areas in Thailand: A survey assessment
Source: PLoS One. 2019 Nov 13;14(11):e0225172. doi: 10.1371/journal.pone.0225172 (PMC6853317; doi:10.1371/journal.pone.0225172)
Supplement: S1 File — (PDF) [file pone.0225172.s001.PDF]

Ethics Committee

Institute for the Development of Human Research Protections (IHRP)

Building 8 Floor 7 Room 702 Department of Medical Science Ministry Public Health Nonthaburi Thailand 11000

Certificate of Approval

Title of Project: Antimicrobial use in Mandarin (*Citrus reticulata* Blanco): use and alternative options for treatment of greening diseases in two major producing areas in Thailand. (Version 2/ Date: April 5, 2017)

Principal Investigator: Sunicha Chanvatik

Responsible Organization: International Health Policy Program Foundation

The Ethics Committee of Institute for the Development of Human Research Protections (IHRP) had reviewed the research proposal. Concerning on scientific, ICH-GCP and ethical issues, the committee has approved for the implementation of the research study mentioned above.

(Dr.Vichai Chokevivat)	(Dr.Pramote Stienrut)
Chairman	Committee and Secretary

Date of First Meeting: March 14, 2017

Date of Approval: April 10, 2017
