# Supplementary material for: Antibiotic use in mandarin production (Citrus reticulata Blanco) in major mandarin-producing areas in Thailand: A survey assessment
Source: PLoS One. 2019 Nov 13;14(11):e0225172. doi: 10.1371/journal.pone.0225172 (PMC6853317; doi:10.1371/journal.pone.0225172)
Supplement: S2 File — (PDF) [file pone.0225172.s002.pdf]

Questionnaire (Antibiotic use in mandarin in 2016)

Interview Date…………………………………..…………………………..

Please complete the following questionnaire by checking the boxes most applicable to you or filling in the blanks

Part 1 General information (code; 99 and 0 = missing)

1. Name..

2. Gender

   (1) Male

   (2) Female

3. Age ....year

4. Status

   (1) Owner

   (2) Cousin

   (3) Employee

   (4) Others please identify……………

5. Highest education level

   (1) Uneducated

   (2) Primary school

   (3) Junior high school

   (4) Senior high school

   (5) Vocational Certificated

   (6) Undergraduate

   (7) Postgraduate

6. Membership in Co-operative relevant to mandarin

   (1) Never

   (2) Yes (please identify).......................

7. Work experience in the production in mandarin orchard ……………………………….…… year

8. Mandarin cultivated area..................Rai

9. No. of mandarin trees in your orchard..................

10. In 2016, do you still growing mandarin?

   (1) Yes (Active farmers in 2016)

   (2) No (Non-active farmers in 2016)

Part 2 Status and management of greening disease during January-December 2016 (code; 99 and 0 = missing)

11. Common diseases in your mandarin orchards (Multiple answers are allowed)

(11.1) Greening disease

(11.2) Root rot

(11.3) Tristeza

(11.4) Scab

(11.5) Black mold disease

(11.6) Tearstain mold disease

(11.7) Canker disease

(11.8) Citrus gummosis

(11.9) Pink mold disease

(11.10) Green mold disease

(11.11) Others (Please identify) (11.11.1)........................

12. How many percentages of mandarin trees that were affected by greening disease (Please tick one answer) **(If answer "Greening disease" to question 11)**

(1) Less than 30%

(2) Between 30% and 50%

(3) More than 50%

(4) More than 100%

13. How to manage greening disease in your mandarin orchards (Multiple answers are allowed) **(If answer "Greening disease" to question 11)**

(13.1) Cut the affected branches without burning

(13.2) Cut and burned the affected branches

(13.3) Partially eradicated the severely affected sections

(13.4) Total eradication

(13.5) Used chemical control

(13.6) Used antibiotics

14. The reason why using antibiotics in management of greening disease (Multiple answers are allowed) **(If answer "Greening disease" to question 11)**

(14.1) Good outcome

(14.2) Low cost, worth investment

(14.3) Hight cost but worth investment

(14.4) Only one choice left

(14.5) Others (Please identify) (14.5.1)..

Part 3 Use of antibiotic during January-December 2016 (code; 99 and 0 = missing)

15. Have you used any antibiotics in mandarin orchards for treatment of greening disease in 2016? (Please tick one answer)

(1) No

(2) Yes (Please give a reason) (15.1)…………………………………………………………

16. Type of antibiotics used in mandarin orchards for treatment of greening disease (Multiple answers are allowed) **(If answer "Yes" to question 15)**

(16.1) Tetracycline

(16.2) Penicillin

(16.3) Ampicillin

(16.4) Amoxycillin

(16.5) Combination antibiotics (Please identify

16.5.1)(1) tetra + ampi (2) ampi +amoxy

(16.6) Others (Please identify) (16.6.1)……………………………………………

17. How to mix antibiotic into the water **(If answer "Yes" to question 15)**

(17.1) **Antibiotic 1**

(17.1.1) Active pharmaceutical ingredient

Powder…………………...gram mix in the water............................L

(17.1.2) Finished product

Tablet: ....mg / 1 tab use...............tab(s) mix in the water..................L

(17.2) **Antibiotic 2 (In case of combination antibiotics)**

(17.2.1) Active pharmaceutical ingredient

Powder…………………...gram mix in the water............................L

(17.2.2) Finished product

Tablet: ....mg / 1 tab use...............tab(s) mix in the water..................L

18. Whom did you get the information about use of antibiotics for greening disease (Please tick one answer) **(If answer "Yes" to question 15)**

(18.1) Neighbouring orchards

(18.2) Farmers in other districts

(18.3) Agrochemical suppliers

(18.4) Pharmacies

(18.5) Pharmacy retailers

(18.6) Academic sector (Please identify) (18.6.1)……………………………………

(18.7) Government sector (Please identify) (18.7.1)...

(18.8) Others (Please identify) (18.8.1)..

19. Source of antibiotics using in your mandarin orchards (Please tick one answer) **(If answer "Yes" to question 15)**

(19.1)☐ Neighbouring orchards

(19.2)☐ Farmers in other districts

(19.3)☐ Agrochemical suppliers

(19.4)☐ Pharmacies

(19.5)☐ Pharmacy retailers

(19.6)☐ Academic sector (Please identify) (19.6.1)……………………………

(19.7)☐ Government sector (Please identify) (19.7.1)...

(19.8)☐ Others (Please identify) (19.8.1)..

20. How to apply antibiotics in mandarin orchards (Multiple answers are allowed) **(If answer "Yes" to question 15)**

(20.1)☐ Pouring at the trunk

(20.2)☐ Spraying on the tree leaf

(20.3)☐ Injection at the tree

(20.4)☐ Others (Please identify) (20.4.1)..................................

21. Which method did you apply of antibiotics injection in mandarin orchards (Please tick one answer) **(If answer "Injection at the tree" to question 20)**

(21.1)☐ Using syringe for injection

(21.2)☐ Dripping

(21.3)☐ Others (Please identify) (21.3.1)..................................

22. Point of injection (Multiple answers are allowed) **(If answer "Injection at the tree" to question 20)**

(22.1)☐ Trunk

(22.2)☐ Main branches showing clinical sign of greening disease

(22.3)☐ branches showing clinical sign of greening disease

(22.4)☐ Others (Please identify) (22.4.1)....................

23. Mandarin age of first injection (Please tick one answer) **(If answer "Injection at the tree" to question 20)**

(1)☐ Less than age of 1 year

(2)☐ Age of 1-2 years

(3)☐ Age of 2 and 3 years

(4)☐ More than age of 3 years

24. Factors affecting volume of injection antibiotics in mandarin orchards (Multiple answers are allowed) **(If answer "Injection at the tree" to question 20)**

(24.1)☐ Age dependent

(24.2)☐ Size dependent

(24.3)☐ Clinical sign of greening disease dependent

(24.4)□ Others (Please identify) (24.4.1)..

25. Frequency of injection antibiotics in mandarin orchards: every.............month(s) **(If answer “Injection at the tree” to question 20)**

(25.1) Small tree: Volume of injection antibiotics................ml/ 1 tree/1 time

(25.2) Large tree: Volume of injection antibiotics................ml/ 1 tree/1 time

26. When to stop antibiotics before harvest (Please tick one answer) **(If answer “Injection at the tree” to question 20)**

(1)□ Less than 1 month

(2)□ More than 1 month

(3)□ More than 2 month

(4)□ Others (Please identify)...................................

………
