# Supplementary material for: Antibiotic use in mandarin production (Citrus reticulata Blanco) in major mandarin-producing areas in Thailand: A survey assessment
Source: PLoS One. 2019 Nov 13;14(11):e0225172. doi: 10.1371/journal.pone.0225172 (PMC6853317; doi:10.1371/journal.pone.0225172)
Supplement: S3 File — (PDF) [file pone.0225172.s003.pdf]

แบบสอบถาม (การใช้ยาปฏิชีวนะในสวนส้มในพ.ศ. 2559)

วันที่สัมภาษณ์………………………………..…………………………..

คำชี้แจง โปรดทำเครื่องหมาย √ ลงในช่อง ☐ หรือ เติมข้อความให้สมบูรณ์

ตอนที่ 1 ข้อมูลทั่วไป (รหัส 99 และ 0 คือ missing)

1. ข้าพเจ้า (นาย/นาง/นางสาว)..

2. เพศ

(1) ☐ ชาย

(2) ☐ หญิง

3. อายุ ....ปี

4. สถานะผู้ตอบแบบสอบถาม

(1) ☐ เจ้าของสวน

(2) ☐ ญาติ

(3) ☐ ลูกจ้าง

(4) ☐ อื่นๆ ระบุ……………

5. ระดับการศึกษาขั้นสูงสุด

(1) ☐ ไม่เรียนหนังสือ

(2) ☐ ประถมศึกษา

(3) ☐ มัธยมศึกษาตอนต้น

(4) ☐ มัธยมศึกษาตอนปลาย

(5) ☐ ปวช./ปวส.

(6) ☐ ปริญญาตรี

(7) ☐ สูงกว่าปริญญาตรี

6. ท่านเป็นสมาชิกกลุ่มที่เกี่ยวข้องกับส้มหรือไม่

(1) ☐ ไม่เป็น

(2) ☐ เป็น (ระบุ)........................

7. ท่านมีประสบการณ์ในการปลูกส้มมาเป็นจำนวนกี่ปี ....ปี

8. เนื้อที่ปลูกส้มโดยประมาณ ....ไร่

9. จำนวนต้นส้มทั้งสวน........................ต้น

10. ปี 2559 ท่านยังทำสวนส้มอยู่หรือไม่

(1) ☐ ทำอยู่

(2) ☐ เลิกแล้ว

ตอนที่ 2 การระบาดและการจัดการโรคกรีนนิ่ง เดือน ม.ค.-ธ.ค. 2559 (**รหัส** 99 **และ** 0 **คือ** missing)

11. สวนส้มของท่านพบโรคอะไรบ้าง (ตอบได้มากกว่า 1 ข้อ)

(11.1) ☐ **กรีนนิ่ง**

(11.2) ☐ รากเน่าโคนเน่า

(11.3) ☐ ทริสเตซ่า

(11.4) ☐ สะแค็บ

(11.5) ☐ ราดำ

(11.6) ☐ ราน้ำหมาก

(11.7) ☐ แคงเกอร์

(11.8) ☐ ยางไหล

(11.9) ☐ ราสีชมพู

(11.10) ☐ ราสีเขียว

(11.11) ☐ อาการผิดปกติอื่นๆ(11.11.1).......................

12. เปอร์เซ็นต์การเป็นโรคกรีนนิ่งครั้งล่าสุด (ตอบได้เพียง 1 ข้อเท่านั้น) **(ถามเฉพาะผู้ที่ตอบ "กรีนนิ่ง" ในข้อ 11)**

(1) ☐ มีต้นส้มเป็นโรค น้อยกว่า 30%

(2) ☐ มีต้นส้มเป็นโรค 30-50%

(3) ☐ มีต้นส้มเป็นโรค มากกว่า 50%

(4) ☐ มีต้นส้มเป็นโรค 100%

13. ท่านมีวิธีการจัดการโรคกรีนนิ่ง (ตอบได้มากกว่า 1 ข้อ) **(ถามเฉพาะผู้ที่ตอบ "กรีนนิ่ง" ในข้อ 11)**

(13.1) ☐ ตัดแต่งกิ่งที่เป็นโรคออกแล้วนำไปทิ้งโดยไม่เผา

(13.2) ☐ ตัดแต่งกิ่งที่เป็นโรคออก และเผาทำลาย

(13.3) ☐ กำจัดต้นส้มที่เป็นโรคทิ้งแล้วปลูกทดแทน

(13.4) ☐ กำจัดต้นส้มทั้งที่เป็นโรคและต้นข้างเคียงที่ไม่เป็นโรคทิ้งทั้งสวน

(13.5) ☐ ใช้สารเคมีป้องกันกำจัดโรคพืช

(13.6) ☐ ใช้ยาปฏิชีวนะ

14. เหตุผลที่เลือกใช้ยาปฏิชีวนะ สำหรับการจัดการโรคกรีนนิ่ง (ตอบได้มากกว่า 1 ข้อ) **(ถามเฉพาะผู้ที่ตอบ "กรีนนิ่ง" ในข้อ 11)**

(14.1) ☐ เป็นวิธีที่ได้ผลดี

(14.2) ☐ ต้นทุนไม่สูงมากนัก คุ้มค่าแก่การลงทุน

(14.3) ☐ ต้นทุนสูง แต่คุ้มค่าแก่การลงทุน

(14.4) ☐ ไม่มีทางเลือกอื่นนอกเหนือจากใช้ยาปฏิชีวนะ

(14.5) ☐ อื่นๆระบุ(14.5.1)..

ตอนที่ 3 การใช้ยาปฏิชีวนะในเดือน ม.ค.-ธ.ค. 2559 (รหัส 99 และ 0 คือ missing)

15. ในสวนส้มของท่าน มีการใช้ยาปฏิชีวนะ หรือไม่ (ตอบได้เพียง 1 ข้อเท่านั้น)

(1) ☐ ไม่ใช้

(2) ☐ ใช้ ระบุเหตุผล (15.1)…………………………………………………………………………

16. ชนิดของยาปฏิชีวนะที่ใช้ (ตอบได้มากกว่า 1 ข้อ) **(ถามเฉพาะผู้ที่ตอบ "ใช้" ในข้อ 15)**

(16.1) ☐ เตตราไซคลิน

(16.2) ☐ เพนนิซิลิน

(16.3) ☐ แอมพิซิลิน

(16.4) ☐ อะม็อกซี่

(16.5) ☐ ผสมกัน ระบุ 16.5.1) (1) tetra + ampi (2) ampi +amoxy

(16.6) ☐ อื่นๆ ระบุ(16.6.1)……………………………………………

17. สัดส่วนการเตรียมยาปฏิชีวนะ (ตอบได้มากกว่า 1 ข้อ) **(ถามเฉพาะผู้ที่ตอบ "ใช้" ในข้อ** 15)

(17.1) ☐ **ยาปฏิชีวนะ ชนิดที่** 1

(17.1.1)☐ผง…………………...กรัม ต่อน้ำ............................ลิตร

(17.1.2)☐เม็ด.................เม็ดๆละ.................มิลลิกรัม ต่อน้ำ..........................ลิตร

(17.2) ☐**ยาปฏิชีวนะ ชนิดที่** 2 **(หากมีการผสมกัน)**

(17.2.1)☐ผง…………………...กรัม ต่อน้ำ............................ลิตร

(17.2.2)☐เม็ด.................เม็ดๆละ.................มิลลิกรัม ต่อน้ำ..........................ลิตร

18. ได้รับคำแนะนำการใช้ยาปฏิชีวนะ จากใคร (ตอบได้เพียง 1 ข้อเท่านั้น) **(ถามเฉพาะผู้ที่ตอบ "ใช้" ในข้อ 15)**

(18.1)☐ เพื่อนบ้าน/สวนข้างเคียง

(18.2)☐ เจ้าของสวนส้มในต่างพื้นที่

(18.3)☐ ร้านขายปุ๋ย/สารเคมี

(18.4)☐ ร้านขายยา

(18.5)☐ ผู้แทนขายยา

(18.6)☐ นักวิชาการ/อาจารย์จากมหาวิทยาลัย ระบุ(18.6.1)……………………………………

(18.7)☐ หน่วยงานราชการ ระบุ(18.7.1)...

(18.8)☐ อื่นๆ ระบุ(18.8.1)..

19. ยาปฏิชีวนะที่นำมาใช้ในสวนส้ม ได้มาจากไหน (ตอบได้เพียง 1 ข้อเท่านั้น) **(ถามเฉพาะผู้ที่ตอบ "ใช้" ในข้อ** 15)

(19.1)☐ เพื่อนบ้าน/สวนข้างเคียง

(19.2)☐ เจ้าของสวนส้มในต่างพื้นที่

(19.3)☐ ร้านขายปุ๋ย/สารเคมี

(19.4)☐ ร้านขายยา

(19.5)☐ ผู้แทนขายยา

(19.6)☐ นักวิชาการ/อาจารย์จากมหาวิทยาลัย ระบุ(19.6.1)……………………………………

(19.7)☐ หน่วยงานราชการ ระบุ(19.7.1)...

(19.8)☐ อื่นๆ ระบุ(19.8.1)..

20. วิธีการใช้ยาปฏิชีวนะ (ตอบได้มากกว่า 1 ข้อ) **(ถามเฉพาะผู้ที่ตอบ "ใช้" ในข้อ 15)**

(20.1) □ ราดลงดิน

(20.2) □ พ่นบนใบ

(20.3) □ ฉีดเข้าลำต้น

(20.4) □ อื่นๆ(20.4.1)..................................

21. วิธีการฉีดยาปฏิชีวนะเข้าลำต้น (ตอบได้มากกว่า 1 ข้อ) **(ถามเฉพาะผู้ที่ตอบ "ฉีดเข้าลำต้น" ในข้อ 20)**

(21.1) □ ใช้เข็มฉีดยาเข้าลำต้นโดยตรง

(21.2) □ ใช้กระบอก หรือขวดพลาสติค ใส่ยา แล้วต่อด้วยสายยางเข้าลำต้น

(21.3) □ อื่นๆ(21.3.1)..................................

22. จุดที่มีการฉีดยาปฏิชีวนะเข้าลำต้น (ตอบได้มากกว่า 1 ข้อ) **(ถามเฉพาะผู้ที่ตอบ "ฉีดเข้าลำต้น" ในข้อ 20)**

(22.1) □ โคนลำต้นหลัก

(22.2) □ กิ่งหลักที่มีอาการ

(22.3) □ กิ่งแขนงที่มีอาการ

(22.4) □ อื่นๆ(22.4.1)..................................

23. อายุเฉลี่ยของต้นส้มที่เริ่มฉีดยาปฏิชีวนะ (ตอบได้เพียง 1 ข้อเท่านั้น) **(ถามเฉพาะผู้ที่ตอบ "ฉีดเข้าลำต้น" ในข้อ 20)**

(1) □ น้อยกว่า 1 ปี

(2) □ 1 -2 ปี

(3) □ 2-3 ปี

(4) □ มากกว่า 3 ปี

24. ปริมาณยาปฏิชีวนะที่ใช้ฉีดต่อ 1 ต้น ขึ้นอยู่กับ (ตอบได้มากกว่า 1 ข้อ) **(ถามเฉพาะผู้ที่ตอบ "ฉีดเข้าลำต้น" ในข้อ 20)**

(24.1) □ อายุต้นส้มมากขึ้น ใช้ยามากขึ้น

(24.2) □ ขนาดต้นส้มใหญ่ขึ้น ใช้ยามากขึ้น

(24.3) □ ระดับความรุนแรงของโรคกรีนนิ่งรุนแรงขึ้น ใช้ยามากขึ้น

(24.4) □ อื่นๆ(24.4.1)..

25. ความถี่ในการฉีดยาปฏิชีวนะ ในปี 2559 ทุกๆ ....เดือน **(ถามเฉพาะผู้ที่ตอบ “ฉีดเข้าลำต้น” ในข้อ 20)**

(25.1) ปริมาณยาปฏิชีวนะที่ฉีดต้นเล็ก................มิลลิลิตร/1 ต้น/1ครั้ง

(25.2) ปริมาณยาปฏิชีวนะที่ฉีดต้นใหญ่................มิลลิลิตร/1 ต้น/1ครั้ง

26. การหยุดฉีดยาปฏิชีวนะก่อนการเก็บเกี่ยว (ตอบได้เพียง 1 ข้อเท่านั้น) **(ถามเฉพาะผู้ที่ตอบ “ฉีดเข้าลำต้น” ในข้อ 20)**

(1) ☐ น้อยกว่า 1 เดือน

(2) ☐ 1 เดือนขึ้นไป

(3) ☐ 2 เดือนขึ้นไป

(4) ☐ อื่นๆ...................................

……
