# Supplementary material for: Antibiotic use in mandarin production (Citrus reticulata Blanco) in major mandarin-producing areas in Thailand: A survey assessment
Source: PLoS One. 2019 Nov 13;14(11):e0225172. doi: 10.1371/journal.pone.0225172 (PMC6853317; doi:10.1371/journal.pone.0225172)
Supplement: Attachment — Submitted filename: Response to editor reviewer as of 2019 10 18 PLOS One.doc [file pone.0225172.s005.doc]

**October 18, 2019**

**Ref.:  PONE-D-19-19263**

**Antibiotic use in mandarin production (*Citrus reticulata* Blanco) in major mandarin-producing areas in Thailand: a survey assessment**

Dear Editor, PLOS ONE,

Many thanks for the feedbacks by two independent reviewers; we found them very useful which strengthens the manuscript significantly. We had fully reviewed and responded to all comments and suggestions; both in track changes and in clean text versions. This file provides detail point by point responses to their comments.

We submit the following:

1. A revised version: clean text version 18 October 2019
2. A track change version showing edits in responses to the reviewers, version 18 October 2019
3. A response to reviewers’ point by point comments (which is this file)
4. Four supporting files:

- S1_file: Ethical approval (word file)
- S2_file: Questionnaire English version (word file)
- S3_file: Questionnaire Thai version (word file)
- S4_file: Raw data for analysis (Excel file)

We look forwards to hearing from you if there is anything we need to take further action.

Best wishes,

Sunicha Chanvatik

On behalf of author team

**Journal Requirements**

Please ensure that your manuscript meets PLOS ONE's style requirements, including those for file naming. The PLOS ONE style templates can be found at

http://www.journals.plos.org/plosone/s/file?id=wjVg/PLOSOne_formatting_sample_main_body.pdf and http://www.journals.plos.org/plosone/s/file?id=ba62/PLOSOne_formatting_sample_title_authors_affiliations.pdf

**RESPONSES**

Many thanks for the advice; the style requirement by PLOS ONE had been fully complied with.

Please include copies of the survey questions or questionnaires used in the study, in both the original language and English, as Supporting Information, or include a citation if they have been published previously.

**RESPONSES**

We had attached four supporting files as followed:

- S1_File: Ethical approval
- S2_File: Questionnaire English version
- S3_File: Questionnaire Thai version
- S4_File: Raw data for analysis

Please ensure that you refer to Figures 1, 2 and 4 in your text as, if accepted, production will need this reference to link the reader to the figures.

**RESPONSES**

In the result section 2. status of greening disease in 2016, we add “Fig 1” at the end of the first paragraph.

…..There were also other fungal diseases with citrus gummosis (18.7%), tristeza (11.9%), and mandarin pests also reported (Fig 1).

Fig 1. Common diseases in surveyed mandarin orchards in Thailand, 2016 (Multiple answers).

In the result section 3. Management of greening disease; at the end of the first paragraph; we add “Fig 2”.

…..33.1% used chemical control; and only 1.8% applied total eradication (Fig 2).

Fig 2. Management of greening disease in surveyed mandarin orchards in Thailand, 2016 (Multiple answers).

In the result section 3.3 Source of antibiotics; at the end of the paragraph we add “Fig 3”.

….. farmers in other districts (6.4%) and academic sector (1.3%). See Fig 3.

Fig 3. Source of antibiotic drugs in mandarin orchards in Thailand, 2016.

In the result section 3.4 Information about use of antibiotics for greening disease, at the end of the paragraph, we add “Fig 4”.

…..and other sources such as advertising on the radio (1.6%) (Fig 4).

Fig 4. Information about using antibiotics in mandarin orchards in Thailand, 2016.

**Comments by the Editor**

One Health platform needs to be defined somewhere in the text along with a listing of benefits it can provide to orchard owners.

**RESPONSES**

We clarified One Health platform in second paragraph of recommendations and provide examples of benefits of One Health solution to the orchard owners.

Secondly, addressing the complex issue such as greening disease requires multi-sectoral, multi-disciplinary collaboration through One Health approach. This includes a) Department of Agriculture, Ministry of Agriculture and Cooperatives responsible for controlling plant disease such as vector control, promotion of low cost disease free propagules replacing mandarin in the affected areas, providing technical support and advices to farmers; b) Food and Drug Administration regulates distribution of antibiotics; and local health centers create awareness of AMR and knowledge about consequences of inappropriate use of antibiotics; c) Department of Environmental Quality Promotion responsible for environment protection from antibiotic contamination in the water and soil can provide advice on safe disposal of antibiotics; and d) International Health Policy Program, of the Ministry of Public Health responsible for health systems and policy research can convene policy dialogues among three sectors: agriculture, health and environment to find best possible solutions. The One Health approach benefits mandarin farmers for example, control of disease and improve productivity without excessive use of antibiotics; increased awareness on AMR and proper use of antibiotics; and protect their environment from the emergence of resistant pathogens. Further, policy decisions should be informed by evidence on whether antibiotics should be registered ….

The audience appears to be policy makers who might read the paper and be led to take action to restrict usage of antimicrobials against HLB or establish greater dialog with stakeholders. Possibly it includes persons involved in extension, development of control methods, or wide-area management, but there is little discussion of past efforts in these areas. Is that because previous efforts have been limited? In that case, you might discuss efforts that have occurred in other countries.

**RESPONSES**

Many thanks for the suggestion. In the discussion section, we add a new sub-section on Management of greening diseases, and propose two new paragraphs as followed

In the past years, the government advised farmers to control greening disease by partial eradication of infected branches or trees and chemical control of vector. The government also supported farmers with disease-free propagules for replacement. However, these control efforts were not successful; as there was no collective and synchronized effort by farmers to control the vectors and removal of affected plants in the whole affected areas; that the vectors move from the non-controlled areas to infect the controlled areas. The abandoned farms with heavily infected trees are the major pool of disease propagation through vectors.

Control efforts in other countries are similar to that applied in Thailand but more intensive and collective. These are vector control, removal of infected trees, providing enhanced nutrition by foliar sprays of readily absorbable nutrients and phytohormones, regulating soil pH to enhance nutrient uptake and precision irrigation using soil moisture sensing to the needs of HLB-affected trees. [1-4].

At line 225, Reference 26 may actually be 25.

**RESPONSES**

Many thanks for the comment, we had edited and done.

**Reviewer 1 Comments to Author**

**General comments**

The manuscript by Chanvatik et al., described the current status of citrus HLB and the utilization of antibiotics to control this disease in two Thailand provinces using questionnaire method. The authors used the appropriate analysis methods. This is a very interesting work. Based on the analysis results, we can know that the citrus HLB and use of antibiotics to control this disease are very common in Thailand, which will lead the AMR concern. The authors pointed out the limitation of this study and discussed the potential sustainable solutions and recommendation for the disease control of citrus HLB in Thailand. I have several minor issues as following.

The data listed in table 1 is mean or median value? Please clarify.

**RESPONSES**

Many thanks for the queries. Data in table 1 showed the number and profiles of respondents in questionnaire survey covered by the study. Some profiles of respondents, such as age and work experience in the orchard, were mean value.

Note that data on dose of antibiotics use in Table 2 are median PPM.

Reference 1 and 6 were repeated.

**RESPONSES**

Many thanks for the comment, we had made corrections.

I suggested the authors provide the raw data for each analysis as supplemental data.

**RESPONSES**

Many thanks for the suggestion, we had provided the raw data of the survey in supplemental file S4 (excel file).

**Reviewer 2 Comments to Author:**

This manuscript reports on the use of a survey of mandarin farmers in Thailand to assess their practices in controlling citrus greening disease. What was found was that antibiotic use was highly prevalent, although no antibiotics were registered for use in disease control.

Apparently, information from neighboring farmers was the main driver in influencing individual farmers to use antibiotics. The survey did uncover sources of antibiotics to the growers, including their ability to obtain active pharmaceutical ingredients (API). One point that was not clear was that the authors listed doses of antibiotics used but I am not sure they are correctly listing these. For example, what is the concentration of ampicillin in API or in pills that are used by the farmers?

**RESPONSES**

Many thanks for the comment and suggestion. Actually, we had provided sources of antibiotics in Section 3.3 on Source of antibiotics where Figure 3 provide different sources of antibiotics accessed by farmers.

The paper also discussed on the ability to obtain active pharmaceutical ingredients (API) in a subsection on “Easy access to antibiotics and weak regulation of API” under the section on discussion. This subsection provide evidence on weak regulation on access to antibiotics which result in easy access by farmers.

For the estimate of doses of antibiotics (PPM) used in mandarin orchards, we asked all respondents on how they mixed antibiotic with tap water. For example,

- If they use antibiotics in API form, they had to fill in below question

Powder…………………...gram mix in the water.............................Litre.

- If they use antibiotics in finished product form such as capsules, they had to fill in this below question

Capsule: ..................mg / 1 capsule, apply...............capsule(s) mix in the water..................Litre.

After that, we can calculate the dosage in PPM for each drug by the plant experts in researcher team.

In the materials and methods section, sub-section on Sampling and Data Collection method section; we clarified as followed.

Dose of antibiotic treatment is measured by part per million (PPM); where 1 ppm equal to 1 mg of antibiotics per litre of water. Farmers are asked a) if they used active ingredient, how many grams of powder were mixed with how many litres of waters; or b) if they used finished products such as capsules, what are the milligram of antibiotics per capsule, how many capsule(s) are added with how many litres of water.

While the results are clearly disturbing, I am not sure if the results of a single survey warrant publication as a full manuscript. Also, it is not clear that the authors are that well versed in agricultural techniques etc. I did not see any statistical analysis of the results. Finally, I am not sure if all of the necessary requirements are present for conducting a survey and utilizing survey information in a publication.

**RESPONSES**

Many thanks for the comment. With limitation of budget; we cannot conduct national survey in all provinces. The researchers do not claim this study represent Thailand as the title of the paper clearly describe the scope of this study “in major mandarin-producing areas” and the method spell out two districts which are the highest density of mandarin orchards.

Descriptive statistics are fully provided in relevant tables. The research received national research ethical review approval. See supplement file S1.

The research team consists of four plant pathologist and plant disease experts; three of them are doctoral in plant pathology. One researcher is expert in Mandarin and doctoral thesis on greening disease.

Ln 50 – probably a brief description and some references for trunk injection are needed as some readers of the manuscript would likely not be familiar with this practice. Also, is there anything known about the relationship between injected chemicals and residues in fruit?

**RESPONSES**

In the fourth paragraph of Introduction section, we provide a brief description of trunk injection technique, and further details and references on trunk injection.

In Thailand, the use of antibiotics in citrus was first piloted in 1998 and then widely practiced for managing greening disease through trunk injection since 2012. For trunk injection, farmers drill hole(s) on the trunk 50-70 cm above the ground, and push by syringes or drip antibiotic solution in plastic bottles into the tree through the hole(s). Trunk injection of a mixture of streptomycin (250 mg/l), ampicillin (2.5 g/l), penicillin G (2 g/l), and Bacicure® (2 g/l) provided the highest efficiency in reducing and suppressing the Las-bacterium population in the field experiments [5]. Injection of 15 syringes per tree of 500 mg ampicillin/20ml water/syringe on pomelo, Koaw-Tang-Kwao cultivar every 2 months reveal high recovery from greening symptom from 4 through 10 months after injection [6]

Lns 96-97 – need more information on the peer review conducted on the survey questionnaire

**RESPONSES**

In the section on Material and method, sub-section on questionnaire survey, we clarified how questionnaire was drafted, peer reviewed, piloted before the final version.

The survey questionnaire, developed in the Thai language, was peer reviewed by two external experts from different fields (pharmacologist and plant expert) for content validity, the logic and clarity of the content. It was pilot tested with 20 farmers, which were randomly selected from orchard growers in two provinces, before the final version was reached with an aim to improve the content validity. We found the contents were easily understood and accurate with no controversy. There was minor amendment of the questionnaire after piloting.

***Table 2 – some of these concentrations seem outrageously high (333,333 ppm?). Do the authors have information on how reliable these estimates are from individual growers? Also, are the antibiotics even soluble at these concentrations? This information should be included.

**RESPONSES**

Many thanks for the comment and a pertinent observation.

We have rechecked and verified this case from the raw data in the questionnaire and had confirmed the result by a phone conversation with the respondent.

This orchard dissolved 1,000 gram of ampicillin of active pharmaceutical ingredients (API) in 3 Litre of water, which is equivalent to 333,333 ppm. In general, beta lactams are water soluble, but the aqueous solubility of ampicillin depends on its salt form and level of pH of solvent. The base form of ampicillin can be dissolved in water at a concentration of 1-10 mg/mL at 70° F (21°C) [7], which is clearly slightly soluble in water and does not make sense that a concentration of 333,333 ppm can be completely dissolved according to OECD's guideline for testing chemicals.

However, given the fact that the most water soluble form can be completely dissolved at 50 mg/mL [8], we have to admit that it is also impossible for such high amount of ampicillin to be completely dissolved.

From our field observation and by talking to the farmers, what we are confident is that such high amount of ampicillin can be dissolved in water to a certain extent. On the contrary, based on limitations, we are not confident on chemical identification of ampicillin (the salt form), the accurate extent to which ampicillin was dissolved, and the solvent, which can affect ampicillin solubility, such as pH.

This point is discussed in a small new paragraph the sub-section on limitation of study of the section on discussion as followed

There is one outlier of very high concentration of antibiotics, up to 333,333 PPM (see Table 2). This concentration is correct after re-confirmation with the respondent. The preparation is 1,000 gram of ampicillin in active pharmaceutical ingredients form in 3 Litres of water; which is equivalent to 333,333 PPM. It is unclear if the reported 1000 gram by the farmer is accurate.

Ln 187 – again, references detailing these techniques should be included.

**RESPONSES**

Many thanks for the comment. In the fourth paragraph of the introduction section, we describe trunk injection technique, as followed:

For trunk injection, farmers drill hole(s) on the trunk 50-70 cm above the ground, and push antibiotics solution by syringes or drip in plastic bottles into the tree through the hole(s). Trunk injection of a mixture of streptomycin (250 mg/l), ampicillin (2.5 g/l), penicillin G (2 g/l), and Bacicure^®^ (2 g/l) provided the highest efficiency in reducing and suppressing the Las-bacterium population in the field experiments [5]. Injection of 15 syringes per tree of 500 mg ampicillin/20ml water/syringe on pomelo, Koaw-Tang-Kwao cultivar every 2 months reveal high recovery from greening symptom from 4 through 10 months after injection [6].

Ln 293 – why is there no recommendation about informing and educating farmers that these practices are illegal and can potentially pollute the environment and influence the occurrence of transferrable antibiotic resistance

**RESPONSES**

Many thanks for the suggestion, we add a few sentences at the end of the first paragraph of recommendation.

Firstly, control of API distribution is needed through effective tracking mechanisms to prevent leakage into agriculture sector. Warning of legal actions for law violations should be issued to retail pharmacies, agrochemical suppliers (which are not licensed to sell any type of medicines) and pharmaceutical retailers selling API directly to consumers. Mandarin farmers should be informed that inappropriate use of antibiotics in mandarin can induce AMR in the pathogens in environment through contamination of antibiotics in water and soil; while at the same time Ministry of Agriculture needs to establish sustainable solution to greening diseases and replace use of antibiotics.

**References**

1.	McCollum G, Baldwin E. Huanglongbing: devastating disease of citrus. 1^st^ ed. Horticultural Review; 2017.

2.	Abdullah TL, Shokrollah H, Sijam K, Abdullah SNA. Control of Huanglongbing (HLB) disease with reference to its occurrence in Malaysia. Afr J Biotechnol. 2018;8: 4007-4015.

3.	Zheng YM, Kumar N, Gonzalez P. Etxeberria E. Strigolactones restore vegetative and reproductive developments in Huanglongbing (HLB) affected, greenhouse-grown citrus trees by modulating carbohydrate distribution. Sci Hortic. 2018;237: 89-95.

4.	Martini X, Pelz-Stelinski KS, Stelinski L. Factors affecting the overwintering abundance of the Asian citrus psyllid (Hemiptera: Liviidae) in Florida citrus (Sapindales: Rutaceae) orchards. Florida Entomol. Soc. 2016;99: 178-186.

5.	Puttamuk T, Zhang S, Duan [Y](https://www.sciencedirect.com/science/article/pii/S0261219414002385#!), antasorn [A](https://www.sciencedirect.com/science/article/pii/S0261219414002385#!), [Thaveecha](https://www.sciencedirect.com/science/article/pii/S0261219414002385#!)i N, Effect of chemical treatments on “Candidatus Liberibacter asiaticus” infected pomelo (Citrus maxcima). Crop Prot. 2014;65: 114-121.

6.	Jaikuankaew S, Paradornuwat A, Chowpongpang S. Huanglongbing disease of pomelo and antibiotics chemotherapy: case study in Koaw-Tang- Kwao from Chainat province, Thailand. The 54th Kasetsart University Annual Conference. Proceeding. 2-5 February 2016. 108-115.

7.	National Center for Biotechnology Information. PubChem Database. Ampicillin, CID=6249, [cited 9 October 2019]. [Internet]. Available from: <https://pubchem.ncbi.nlm.nih.gov/compound/Ampicillin>.

8.	Merck. A9518 - Ampicillin sodium salt. [cited 9 October 2019]. [Internet]. Available from: https://www.sigmaaldrich.com/catalog/product/sial/a9518?lang=en&region=TH.
